# Supplementary material for: Effects of Gluten on Gut Microbiota in Patients with Gastrointestinal Disorders, Migraine, and Dermatitis
Source: Nutrients. 2024 Apr 20;16(8):1228. doi: 10.3390/nu16081228 (PMC11053402; doi:10.3390/nu16081228)
Supplement: Supplementary file 1 [file nutrients-16-01228-s001.zip › nutrients-2920349-supplementary.pdf]

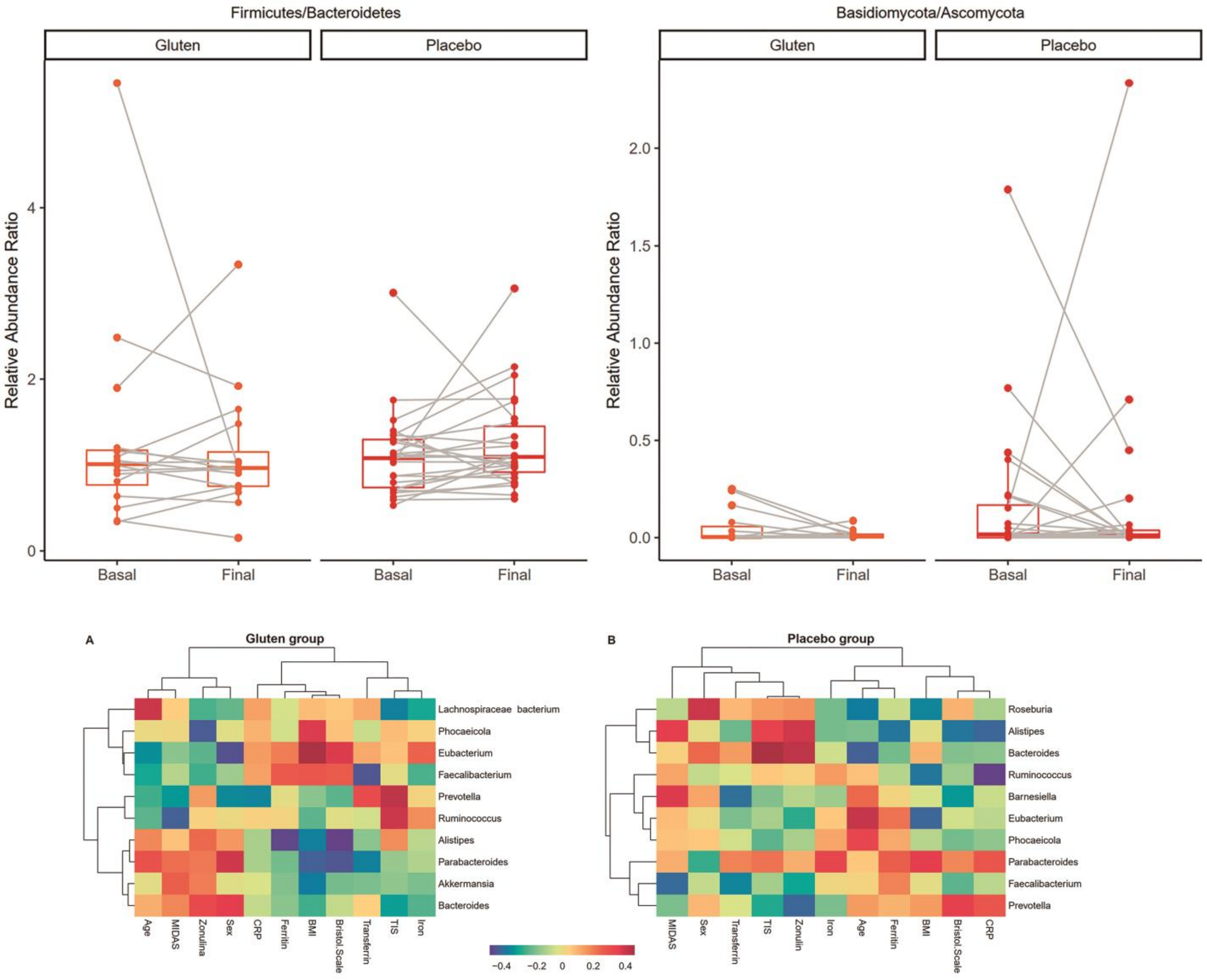

Supplementary Figure S1. Relative abundance of bacteria and correlations between bacteria and study variables **(A) - top -** Differences between the Fimicutes/Bacteroidetes ratio between the 2 times (baseline-final) between both study groups -gluten and placebo-, measured in relative abundance; **(B) – top -** Differences between the Basidiomycota/Ascomycota ratio between the baseline-final and between both study groups, measured in relative abundance. **(A, B) - in the lower area -** Hierarchical clustering of correlations using the Spearman's test between bacterial genera (bottom) and study variables (right side) in the gluten and placebo group.

Supplementary Table S1. Migraine symptoms by time according to study group and with professional diagnosis of migraine.

	Gluten group (n = 12)			Placebo group (n = 12)			Δ Intergroup analysis
**Migraine**	**Basal**	**Final**	***P***	**Basal**	**Final**	***p***	
Pain (0–10)	8 (6-8)	7 (6-8)	.754	6.33 ± 2.46	5.83 ± 2.13	.600	**Ns**
Days with pain (3 months)	7.5 (5.5-40.5)	3 (1.5-13.5)	.109	31.17 ± 30.02	11.83 ± 13.11	**.037**	**Ns**
MIDAS	26 (11.5-61.5)	4.5 (0-40.5)	.388	57.33 ± 53.58	16.25 ± 33.65	**.019**	**Ns**

MIDAS, Migraine Disability Assessment; NS, not significant.

Supplementary Table S2. Symptoms of atopic dermatitis by time according to the study group and with professional diagnosis of dermatitis.

	Gluten group (n = 6)			Placebo group (n = 14)			Δ Intergroup Analysis
**Atopic dermatitis**	**Basal**	**Final**	***P***	**Basal**	**Final**	***p***	
Itchy skin (0–10)	3.5 ± 4.18	2.33 ± 3.67	.135	6.07 ± 2.24	3 ± 3.31	**.001**	**Ns**
Itchy scalp (0–10)	0 (0-0)	0 (0-0)	.317	2.5 (0-8)	0 (0-6)	**.031**	**Ns**
Dry skin intensity (0–10)	1.5 (0-4)	0 (0-5)	.655	6.86 ± 2.6 7.5 (6-8)	3 ± 3.35 1.5 (0-7)	**.001**	**Ns**
Pimples (0–10)	0 (0-0)	0 (0-0)	.317	0 (0-5)	0 (0-0)	.063	**Ns**
Neck and chest area (0–10)	0 (0-0)	0 (0-0)	.317	0 (0-5)	0 (0-0)	**.031**	**Ns**
Face (0–10)	0 (0-0)	0 (0-0)	.317	0 (0-6)	0 (0-0)	.375	**Ns**
Arms and legs (0–10)	0 (0-0)	0 (0-0)	.317	0 (0-8)	0 (0-0)	.063	**Ns**
Whole body (0–10)	0 (0-0)	0 (0-0)	.317	2.5 (0-8)	0 (0-0)	**.018**	**Ns**
TIS	0.5 (0-2)	0 (0-1)	.655	2.93 ± 2.27	1.71 ± 1.73	.051	**Ns**

TIS, Three Item Severity score.

Supplementary Table S3. Iron metabolism, proteins, intestinal permeability, white, red and platelet series by time according to the study group.

	**Gluten group (n = 17)**			**Placebo group (n = 27)**			**Δ Intergroup analysis**
**Iron metabolism**	**Basal**	**Final**	***p***	**Basal**	**Final**	***p***	
Iron (μg/dL)	92.94 ± 43.97	90.18 ± 39.12	.772	88.70 ± 34.83	79.57 ± 39.23	.267	**ns**
Ferritin (ng/mL)	57 (31-131)	63 (25-133)	.454	59 (23-93.5)	70 (28-111.5)	.248	**ns**
Transferrin (mg/dL)	234 (222-254)	251 (215-277)	.210	240 (222.5-265.5)	244 (222.5-262.5)	**.029**	**ns**
**Proteins**							
C-Reactive Protein (mg/dL)	0.10 (0.05-0.20)	0.10 (0.05-0.10)	.424	0.1 (0.1-0.35)	0.1 (0.1-0.25)	1	**ns**
**Intestinal permeability**							
Zonulin (ng/mL)	90.26 ± 51.8	89.66 ± 55.24	.963	111.74 ± 74.23	97.03 ± 61.95	.401	**ns**
**White blood cells**							
Leukocytes	6 (4.6-7)	5.7 (4.5-6.7)	.332	5.9 (5.25-7.4)	6.25 (5.25-7.65)	.572	**ns**
Neutrophils	58.1 (55.4-60.4)	55.2 (51.1-60.4)	.909	56.45 (51.65-61)	55.5 (51.55-59.75)	.792	**ns**
Lymphocytes	31.2 (28.5-33.9)	33.5 (29.4-36.5)	.332	33.15 (29.15-35.9)	32.6 (27.05-35.9)	.531	**ns**
Monocytes	7.8 (7.1-9.4)	7.8 (7.4-9.6)	.791	7.45 (6.45-9.4)	7.45 (6.2-9.15)	.664	**ns**
Eosinophils	2.1 (1.4-3.1)	2.1 (1.4-2.6)	.607	1.95 (1.55-3.1)	1.95 (1.75-3)	.701	**ns**
Basophils	0.7 (0.5-0.8)	0.7 (0.5-0.8)	.534	0.6 (0.4-0.9)	0.6 (0.5-0.75)	.526	**Ns**

Absolute neutrophils	3.4 (2.5-4)	3.2 (2.8-3.6)	.607	3.45 (2.65-4.15)	3.55 (2.8-4.45)	.087	**Ns**
Absolute lymphocytes	1.8 (1.3-1.9)	1.7 (1.5-2.1)	.454	1.95 (1.65-2.4)	1.9 (1.7-2.3)	.738	**Ns**
Absolute monocytes	0.5 (0.4-0.5)	0.5 (0.4-0.5)	.491	0.5 (0.4-0.6)	0.5 (0.4-0.6)	.648	**Ns**
Absolute eosinophils	0.1 (0.1-0.2)	0.1 (0.1-0.1)	1	0.1 (0.1-0.2)	0.1 (0.1-0.2)	.791	**Ns**
Absolute basophils	0.1 (0-0.1)	0 (0-0.1)	.125	0 (0-0.1)	0 (0-0.1)	1	**Ns**
**Red blood cells**							
Red blood cells	4.4 (4.2-5)	4.6 (4.2-4.9)	.969	4.5 (4.2-4.75)	4.5 (4.3-4.85)	.218	**Ns**
Hemoglobin	13.5 (13-15)	14 (12.9-14.6)	.956	13.65 (12.7-14.35)	13.7 (12.65-14.2)	.269	**Ns**
Hematocrit	40.8 (38.9-45.7)	41.6 (39.5-44.1)	.964	41 (37.85-42.65)	40.5 (38-42.55)	.406	**Ns**
MCV	89.5 (87.8-93.5)	89.9 (88.5-92.8)	.844	90.35 (87.9-93.25)	90.9 (88.4-92.9)	.359	**Ns**
MCH	30.4 (29.3-31.1)	30.1 (29.1-31.2)	.605	30.15 (28.95-31.8)	30.45 (29-31.6)	.269	**Ns**
MCHC	33 (32.4-34)	33.2 (32.8-33.5)	.938	33.4 (32.75-34)	33.45 (32.9-34.15)	.838	**Ns**
RDW	13 (12.4-13.5)	13 (12.5-13.2)	.607	12.85 (12.4-13.25)	12.65 (12.3-13.35)	.237	**Ns**
**Platelets**							
Platelets	235 (186-262)	220 (197-252)	.332	266.5 (227.5-290.5)	260 (235.5-276.5)	1	**Ns**

MPV	11.6 (11.3-12.2)	11.8 (11.1-12.4)	.321	11.25 (10.6-12.05)	11.3 (10.55-12)	.786	**Ns**

MCV, mean corpuscular volume; MCH, mean corpuscular hemoglobin; MCHC, mean corpuscular hemoglobin concentration; RDW, red cell distribution width; MPV, mean platelet volume.
